# American Dental Association

**Published:** 1876-01

**Authors:** 


### ARTICLE IV.

### *The American Dental Association—Fifteenth Annual Session.*

FOURTH DAY—*Morning Session.*

The association was called to order at the usual time, the vice-president in the chair. After unimportant routine business, the subject of "Mechanical Dentistry" was taken up, and a report on that subject was read by Dr. Rehwinkel. We give a synopsis, as follows:

The report alluded to the apathy shown in reference to this branch of the profession, and the disposition on the part of some to separate it entirely from the operative department; a state of things which is deemed to be due to the fact that cheap bases have taken the place of gold, and to the want of fostering care on the part of societies. The disgust of the best men with this branch was deprecated, and it was inferred that ere long a formal question as to separation will be proposed, which it is to be hoped will not be hastily decided.

The committee deemed a porcelain plate the highest form of art in this department, although its use must necessarily be limited to a few experts; next to this was placed continuous gum, which is more practical. If not prepared to complete the process himself, the dentist can send it to those accustomed to the furnace, and this plan produces excellent results. That this work is not more demanded is the fault of the dentist; for any reputable practitioner has influence enough to introduce it. A combination of gold and rubber or celluloid stands next in rank, combining the advantages of each. Aluminum has not proved as useful as was hoped. Cheoplasty has never obtained popularity, and, though successfully used, does not seem likely to be *the* substitute for rubber. Watt's and Talbot's metals were spoken of as most likely to be valuable, and the method employed by the latter gives most satisfactory results. The improvements in celluloid were next spoken of, and the committee were glad to say that it has ceased to be an experiment, promising to supersede all other cheap bases. Its advantages—its beauty and lightness, and the absence of injurious effects upon the mouth—were spoken of, as well as the opportunity it afforded for using plain teeth. One member of the committee had worn a plate of celluloid for more than three years which, though of the first material and made by a novice in the work, has stood well to this day. The steam and dry heat methods of working were mentioned, and the last named spoken of as rapidly gaining favor, and possessing advantages, especially in regard to the welding of different pieces, and repairing.

The experiments of D. M. Lamb, of London, Canada who has succeeded in obtaining a vulcanizable gum from milk-weed,—which it is claimed is a successful substitute for rubber—were referred to.

The subject being open for discussion,—

Dr. Stockton said that he had been gratified at the reference to continuous gum. No work is comparable to it. Our colleges turn out young men entirely ignorant of this work. Platinum and iridium, in connection with celluloid, is stronger than gold, and makes excellent work. The dry-heat process of working celluloid makes the hardest and best work. One advantage of it is, that it does not require so close attention as the other methods; and, if necessary, it can be left for an hour or two without injury.

Dr. D. D. Smith. This subject is so kept in the background that there is little encouragement to speak upon it. Operative dentistry only seemed worthy to be discussed and yet there are failures in that department, and patients are continually losing their teeth and being thrown upon the resources of the mechanical department. The two branches cannot be separated, at least in our day. The speaker then presented a set of teeth from one of the best operators in the country, in the construction of which every principal of mechanical dentistry had been violated, and proceeded to point out the manner in which many principles are violated by those who attempt mechanical practice. He spoke of the preparation of the mouth, arrangement of the teeth, and the manner of articulating them. If only one tooth remains in the mouth, with no antagonist, the first principle should be to extract it. The teeth should never be arranged in a horse-shoe shape, and the articulation should be such that the pressure would be equally distributed upon all the teeth.

We have fallen down and worshiped at the shrine of rubber until progress has nearly vanished. Indolence and a love of gain join hands. A few discard it; but the saddest part of it is that only that exactions of the Rubber Company seem to be taken into account in our discussions. If rubber king, then let us bow to law; if it is the material of all others, let us claim and use it; but if not, then let us abandon it for what is better. The Philadelphia Dental College teaches continuous-gum work and metal more than any other; it ignores rubber as far as possible; we cannot, however, entirely control the matter.

Dr. Abbott said that the New York College also taught this work, and he presumes every college does so.

Dr. Atkinson is astonished at the records of the colleges which show more rubber work than any other.

Dr. McQuillen said our demonstrators are instructed to advise patients who come to the Dispensary for artificial teeth to have them on metal plates, but we cannot compel them to do so when they demand the rubber base; nor are students disposed to do more of metal work than they can help.

Dr. Atkinson said they could compel them to deposit a case of continuous-gum work.

Dr. D. D. Smith. Every candidate for graduation in the Philadelphia Dental College is required to show me a set of teeth in the mouth on a metal base, and to present a similar set for the museum. Sets of continuous gum work are deposited in the museum every year.

Dr. Barrett spoke in regard to the new milk-weed-rubber base. It was plain that some cheap base was required, and this seemed to be the thing, and he believed it would prove a perfect substitute for the ordinary rubber. It was an original discovery, being harder, denser, and, he believes, stronger than caoutchouc. It will not inflame, and is a poor conductor. The company only awaited financial arrangements to place it upon the market.

The subject was then passed, and the Committee on Instruments and Appliances made a report, which was accepted.

### INSTRUMENTS AND APPLIANCES.

The following is the substance of the committee's report. Owing to the multiplicity of such articles, and want of time for further detail, the report merely mentioned the articles by name, and gave in some cases the points claimed by the inventors.

Motive-Powers. The Bastet magnetic and the Backus water motors were exhibited.

Dental Engines. Samuel S. White exhibited the S. S. W. engine, also an extension treadle, and an improved hand-piece for engines, the bearings being of hardened steel and far more durable than those made at first.

There were also shown Bonwill's engine, the Elliott suspension engine, Edson & Evans portable suspension engine.

ENGINE PLUGGERS. Samuel S. White exhibited Dr. Buckinham's plugger adapted to the S S. White engine.

Bonwill's electro-magnetic plugger, Gaylord's pneumatic and Swartley's automatic pluggers were also exhibited.

HAND PLUGGERS. Samuel S. White exhibited Dr. Corydon Palmer's complete set of plugging instruments.

Dr. Brockway's smooth-pointed set were also mentioned.

BRACKETS. Samuel S. White presented his improved bracket.

Johnston Bros., exhibited a bracket table.

CHAIRS, ETC. Samuel S. White showed an improvement on the Harris chair, by which the upper section of the back could be quickly removed, thus shortening the back sufficiently for small children ; also a detachable arm for this chair, which may be taken off and replaced readily ; also an improved foot-board.

G. W. Archer exhibited a chair with a compensating spring, which is wound up as the chair is lowered, and expends the accumulated force in a lifting power.

The B. D. M. Co. exhibited Dr. Hayes's new chair.

Dr. O. C. White exhibited his improved chair with spittoon and bracket.

Codman & Shurtleff exhibited Dr. Cogswell's dental cabinet, which was spoken of as compact and perfectly adapted to the purpose for which it was designed.

Johnston Bros. presented a locking device for the castors of the Morrison chair.

LATHES. Samuel S. White exhibited his new office lathes and laboratory lathes, which were very neat.

VULCANIZERS. The Ransom vulcanizer was shown as combining a vulcanizer and a celluloid packer.

S. B. Brown also exhibited a celluloid dry-heat apparatus.

G. E. Hays presented a celluloid packer which could be used in a vulcanizer.

MISCELLANEOUS. Samuel S. White exhibited—

A dentist's lamp and warm water cup, with foil heater and annealer Dr. Butler's instruments for inserting screws. An improved hot-air syringe, with metal guard for the purpose of giving firm support in the palm of the hand while compressing the bulb with the fingers.

Dr. Hickman's double-lipped rubber dam clamp. Dr. Osmond's clamp mirror. Bowman-Allan's clamp forceps. Dr. Bogue's tape forceps. Dr. Frank Abbott's improved back-action engine. A device invented by Dr. G. H. Cushing, for rotating disks at an angle, was highly spoken of. Dr. Klump's porte polisher for engine. Dr. Huey's improved screw-head mandrel, which substitutes a screw for the ordinary nut in mounting disks. Dr. Butler's corundum points for polishing, and a bur cut like a bastard file.

Codman & Shurtleff exhibited—

A simple and cheap tape carrier, a file carrier, and a mouth mirror of improved construction. An ether and gas inhaler, which was highly spoken of.

Johnston Bros. exhibited—

A dental factotum. An improved gasometer for liquid or ordinary gas. Jarvis's separators.

R. S. Williams showed a device for holding gold cylinders, a morocco case containing glass vials labeled.

Dr. Horton's rubber pouch or bib was shown, designed to protect the clothing of the patient.

The subject of "Dental Education" was then taken up, and opened by the reading of a report by Dr. Keely, the chairman of the committee.

The substance of Dr. Keely's paper is as follows:

The future of dentistry depends upon the efforts now making for the elevation of the standard of professional education. Recognition by the medical profession as a specialty, though desirable, is not indispensable. Dentistry demands a high tone of manhood, thorough discipline of the powers, and a careful and elaborate training. Many of the lights of the profession have not enjoyed such education; by effort and ability they have achieved success in spite of this, but they would have the future members of the profession thoroughly furnished. To assume to practice at the present day without better preparation than the older practitioners, argues a failure to comprehend obligations, or an indifference to them. In the practice of dentistry, the truths of science must be embodied. The ideal and the practical must be brought into combination. In our discussions some lay too much stress upon scientific indoctrination, and others overvalue practical skill. A system of education must be inaugurated which shall harmonize these tendencies. As to the ends to be attained, there is unanimity; but as to the methods, there are various opinions. How shall we be relieved from the dead weight which embarrasses us? Shall it be by penal enactments of the civil power, or shall the profession refuse fellowship with any but the worthy and qualified? and what tests of ability shall be prescribed? How shall the fundamental medical and other knowledge be acquired? shall medical colleges recognize the specialty, and teach it, or shall the dental college call to its aid the medical professor to supply the needed basic culture? Both these methods may be tried, but the dental colleges will, of course, be continued. A question of great importance is, whether our efforts in this direction shall be concentrated on a few, or frittered away on a multitude of starvelings that disgrace the profession, and multiply the half-taught aspirants for honors. The example of literary institutions is before us. Want of resources and competition make their honors worthless, and professional occupations are crowded with incompetents. In our branch three first-class colleges only are needed at present, and this number could be endowed, the ablest professors secured, and ample apparatus and libraries provided. If, however, we attempt to sustain a large number of schools, some will succumb to financial pressure, and leave debts and exasperated feelings behind them; and those remaining will be neither healthy nor vigorous as they might have been. This association should inaugurate an effort to accomplish this concentration.

Dr. Flagg then read a volunteer paper on the same subject.

The subject being open for discussion,—

Prof. Shephard said that he questioned the assumptions made by the last reader (Prof. Flagg,) as to his knowledge of the workings of all the colleges of the country. By his own statements it appears that he is not practically familiar with all. In Harvard College there is no subordination of the dental to the medical, or any other department. They are on a par. They may vary in the estimation in which they are held by the community, but so far as the governing body is concerned they are equal, separate, and independent. It is proper and economical that one man should be connected with both facilities. It is our disgrace that dental colleges have not been endowed. The professors give their services, and find their own museum and apparatus. If the state or a university will furnish apparatus, it should be welcomed, and it should not be charged that we are snubbed or tacked on to the tail of any other institution. The abbreviation D.D.S., though thirty years old, is not to be found in any dictionary, because lexicographers derive their authority from classical institutions, and not from special schools. Time will tell which system will turn out the best rounded men.

Dr. McQuillen would approach the consideration of the subject in a dispassionate manner, independent of the interests of any institution, and in favor of the broadest culture, such as is the distinguishing characteristic of an educated gentleman in any walk of life. He believed in the law of evolution as applicable to the individual man, society, professions, and institutions of learning. Fifty years ago there were only three hundred dentists in our country; many of these had failed in other occupations, and took up with this as an easy means of gaining a living. The artisan came from his trade, the blacksmith from his anvil, the carpenter from his jack-plane and saw, and with little or no preparation entered upon practice. A few only had studied medicine, and, dissatisfied with the condition of the craft, conceived the idea of elevating the standard by establishing departments of dentistry in medical colleges. How were they received? So far from welcoming them, the faculties gave the cold shoulder to the proposition. There was only one source left,—to establish institutions where dental students could receive culture, if only a partial one. When it is considered how much there is to know, it must be recognized that the most learned are only partially educated. The first dental college met with only a limited success. Its faculty, composed of medical graduates, sent representatives to the American Medical Association, and these delegates were treated as the applicants for dental departments had been: the cold shoulder was given them. The speaker then quoted from the printed transactions of the American Medical Association for 1852, in support of his statements. Notwithstanding this experience, if a better course of instruction could now be secured by connection with medical schools, he would not stand in the way, But the question is, Can that be done? In dental colleges theory and practice go hand by hand. But the same facility is not found in the medical schools, indeed, cannot be granted in the general practice of medicine and surgery: and it may be that the dental departments would be equally defective in practical instruction. Medical graduates go out to learn the art of medicine, and woe to the community where the tyro has to practice! The graduate of the dental college is to a certain degree prepared to practice.

Just criticisms of the defects of colleges cannot be objected to, for out of such criticisms good will come; but the inuendoes and false statements that so often appear in regard to them are as unjust as they are ungenerous. Years ago Captain Marryatt, Mrs. Trollope, and a host of other writers of the same ilk overran our country, and, returning to their home in England, wrote works in which they misrepresented and ridiculed our country, its people, government, and institutions, and predicted the speedy downfall of the young republic. Not recognizing that in a young country the efforts of man must first be directed towards securing the necessities of life, the felling of trees, the erection of homes, cultivating the land, etc., and that it is not possible for the arts, sciences, and great institutions of learning to be developed at once,—with none of that charity which is ever characteristic of a generous mind, or that broad generalization which belongs to a great one, they could only perceive and exaggerate defects, and could not recognize the possibilities of the great future which was in store for our country and its institutions. Is it surprising that this class of narrow minds should have had a representative in the dental profession, who, visiting our country, making hasty and imperfect examinations of our dental colleges, on returning to his country, in trying to write them down, only exposed his own ignorance and prejudices? We can pity this manifestation of poor, weak, human nature, and regret that in place of trying to degrade and lower the institutions of another country, he did not rather aim to make for the institution with which he is connected a world-wide reputation by proving himself to the profession an able writer and efficient teacher. But what shall be said of an American correspondent of a foreign journal, who, not having the manhood to write over his own signature, Iago-like, in assailing the fair fame and impugning the motives of others, proves that the suspicious man may be ever justly suspected? When such men are dead and forgotten, the institutions which they have reviled will be rendering valuable services to the profession and to humanity. Communications such as the "Chronicles of the Odontologues" place the profession in America in anything but an enviable position, particularly when a British journal apologizes for republishing such articles, and accounts for the appearance of them to the defective development of our people. If the dental colleges are not what they ought to be, sweep them out of existence. If they fail to properly prepare the student for practice, then close them up. The all-important question is, not whether the student receives an education which will entitle him to recognition on the part of medical men, but whether *he is properly fitted to go forth and serve the community as a practitioner of dentistry.* If dental colleges do this, and he believed they do, they should be encouraged and supported. The best evidence of the value of dental colleges is to be found in the leading position of their graduates at home and abroad.

Dr. Atkinson. We should not dwell so much on methods as results. Foundation principles we must have, but they are of no use till they are practicalized. He prefers dentists to medical men. It is easier to define what is required of the dentists.

Dr. Abbott wishes to defend dental colleges,—the independent schools. He referred to a charge made in Canada that the quacks came from the United States, and that the degree was unworthily granted here. He said the New York College turns out as good men as any other college, and he thought the faculty would compare with any.

Dr. Flagg thought the feeling that had arisen, if allowed to continue, would split the profession. He considered that we were able to hold our own, and we should see to it that we do not go to the wall.

The subject was then passed, and that of "Dental Literature" taken up. The chairman of the committee on that subject, Dr. I. Knapp, read a report, of which we give a synopsis.

The report adverted to the fact that dental literature is creditable to a profession that scarcely a third of a century ago began to study scientific principles; before that date, or even later, a single volume was sufficient to describe all then known to the profession. The field, however, is constantly widening, and the time has come for a full discussion of many subjects from a purely dental aspect.

The report then proceeded to review at some length Salter's work on Dental Pathology and Surgery, lately published, and quoted somewhat freely from it, to illustrate the style of the author, and touched upon his theories of development of dentine. The example of the author in giving only the results of original thought and investigation, instead of a compilation, was commended. His style was stated to be simple, and clear, vigorous; a familiarity with scientific terms being manifest, though they are sparingly made use of. His descriptive powers are good, and it is scarcely possible to mistake him, the salient points alone being seized and brought out. A philosophic tone is preserved and conclusions are stated with judicial candor. The author's views of the histology of dentine were then shown by extracts; he takes the ground that the broad, brilliant ring seen in transverse microscopical sections is an optical illusion; upon the formative pulp is arranged a series of columnar cells, from the distal extremities of which minute tubular threads project inwardly, which are gradually prolonged in the same direction, and separated by a homogeneous blastema; these being the only discovered elements in the formation of dentine. The supposed "fibrils of soft tissue" are the decalcified tubes themselves. The indurated viscid contents of the tubes might easily be mistaken for fibrils. It is deemed highly improbable that the nervous elements distributed to the dentine pierce the walls and occupy the axis of the tube.

The report then proceeded to discuss the theories of Tomes, Wedl, and other authors on the same subject, and further quoted Salter upon the subject of secondary dentine. An extract was quoted in reference to a singular case of crushing the inferior dental nerve, in extracting the dens sapientiæ, which resulted in the paralysis of the teeth, lip. and chin of the right side. The author of the report stated that a similar case had come under his own observation, The sensation had never been fully restored.

The work of Dr. Salter, who does not allow himself to be drawn into the discussion of subjects not immediately pertaining to dental pathology and surgery, and therefore says but little in reference to dentistry proper, is a valuable text-book for students and the more advanced cannot fail to find in it both pleasure and profit.

The report then briefly alluded to the periodical literature of the profession. The readiness of the proprietors of the journals to admit a mass of undigested and often irrelevant matter was regretted, but this was considered unavoidable in view of the fact that the editors were largely engaged in other duties, and receive but a limited compensation for editorial work; they can therefore not be expected to reject articles needed to make up the numbers, or to remodel communications, or prepare original matter Still, it was stated that fault was justly found, it is feared, with the neglect or imperfect performance of that editorial work which is needed to prevent serious misapprehension of a writer's or speaker's opinions. The journals on the whole were commended as having done a great and good work, second to no other agency. The establishment of a quarterly journal, edited by a writer of varied and profound learning, giving his whole time to the work, and aided by paid articles, would be a long step in the right direction; not interfering with the other journals, but gathering up that which is valuable now scattered through our transient literature, and affording the best minds of the profession a medium for their best thoughts.

The subject of "Pathology and Surgery" was taken up, and, there being no reports,

Dr. Atkinson said that he had gone further into the change from physiological to pathological action than any other, so far as he had read. Inflammation is the process of disease; the vaso-motor nerves keep up the function, and if the blood is equally distributed, nutrition takes place within the capillaries. When sufficient destruction of neurine has dominion, congestion ensues, and circulation is arrested and remanded back to cell-action, and the fluids are thus changed to soft-solids. That is one termination of the deflected function; another is tuberculous deposits. These are inside the inflammatory process. Disintegration continued generates gases, and the absorption of these converts the blood-corpuscles into pus-corpuscles, which are mid-way between building up and tearing down. Then we get corrosion,—ichor, evidence of death, sloughing. The speaker then spoke of the treatment of fever soies, which he would treat with a solution of chloride of zinc, twenty or forty grains to the ounce, and so get union by first intention. We must know what the tissues are on which we are operating, and what they will bear. Every man's work is either worship or blasphemy. There is no such thing asasthenic disease; to talk of it is asthenic nonsense.

Dr. Taft then spoke of the treatment of diseased gums, produced by calcareous deposits on the necks of the teeth. By merely removing these deposits the trouble will not always be remedied, nor will medicaments always relieve it.

Rubbing is beneficial, pressing out the contents of the capillaries, and they will soon take on a better circulation. He regarded salicylic acid as better than chloride of zinc; aromatic sulphuric acid is also useful. Depletion will be beneficial until fresh blood has taken the place of stagnant blood.

The subject was then passed.

The Committee on Nominations made the following report as the standing committees for the ensuing year, which on motion, was concurred in:

*Dental Physiology.*—J. H. McQuillen, S. W. Dennis, A. H. Brockway.

*Pathology.*—J. Foster Flagg, L. D. Shepard, I. Knapp.

*Chemistry.*—S. B. Palmer, J. S. Cassidy, L. G. Noel.

*Therapeutics.*—W. H. Atkinson, C. A. Brackett, C. R. Butler.

*Operative Dentistry.*—Frank Abbott, A. W. Harlan, H. M. Reid.

*Mechanical Dentistry.*—W. N. Morrison, D. D. Smith, W. C. Barrett.

*Etiology.*—D. C. Hawxhurst, M. H. Webb, H. L. Sage.

*Dental Literature.*—M. S. Dean, E. D. Gaylord, G. C. Daboll.

*Dental Education.*—W. H. Morgan, J. N. Crouse, M. Wells.

*Prize Essays.*—F. H. Rehwinkel, G. W. Keely, H. J. McKellops.

The committee appointed to prepare resolutions on the death of Dr. Hill, of Norwalk, reported the following, which on motion, were adopted ;

*Whereas*, This association has with sorrow learned of the death of our brother, Dr. Asa Hill, of Norwalk, Connecticut ; therefore,

Resolved, That we signify in this public manner our sense of the great loss which the dental profession has sustained, and that we sincerely sympathize with his family in their loss.

*Resolved*, That this resolution be inserted in our minutes and that the secretary be instructed to forward a copy of the same to the family of the deceased.

J. H. Smith,<br>

Frank Abbott, } *Committee.*<br>

A. W. Harlan,

### *Afternoon Session.*

The association met at the hour fixed, four P. M. There being no essays on the subject of "Etiology," that subject was passed and "Therapeutics" taken up.

Dr. Atkinson spoke on the subject of salicylic acid. He said that all concurred in regard to its non-corrosiveness. He dissolves in alcohol to saturation for dressing abscesses and wiping out cavities. It supersedes creasote, and does not destroy the epithelium. It is also useful for stopping hemorrhages. It is nearly inodorous, and also nearly insipid. A solution of the alcoholic tincture in water was a good disinfecting mouth wash and deodorizer of the spittoon. It is also useful as an ingredient in dentifrices, arresting the formation of gases. There was sometimes a disposition to criticise the earnest seeker after truth. He had been enamored with different agents. He had been the first to use creasote in teeth, and had never seen evil results from it in the hands of a level-headed man ; he had heard of cases, but would question the manipulation rather than the agent. He knows it is a deadly poison. His first acquaintance with it was in dyspepsia, an aqueous solution, three or four drops to the ounce, and he don't go back on it now, though he has used thymol, and now uses salicylic acid. Bad results are often from want of management; there is a great deal in manipulation, and there is such a thing as want of compatible tension between the operator and the medicine. In salicylic acid we have pretty nearly chased the active principle of creasote into a corner.

Dr. Taft said he had used salicylic acid with great success for some months, and almost every day in some new phase. In cases of chronic inflammation difficult to reduce he had found it useful. In one tooth which had been filled temporarily for eight years, and had alway been accompanied with some soreness, he had cleansed the canal and filled half full with crystals of salicylic acid and the remainder with oxychloride, and the next morning it was better than for ten years, and the soreness had not since returned. He prefers a saturated solution in ether, which dissolves three times as much as water or alcohol. In roots which are exceedingly offensive there was no odor in a few days. He prizes it, and is doing with it what creasote never would accomplish. Has used it for abscesses with similar results. Its great value lies in its antiseptic properties; it decomposes offensive matter. There is no whitening of the tissues as with creasote. It is not necessary that it should be colorless to be efficient. He had used the "dental pain obtunder" with general good results, and had used it several times a day. With it he can cut without pain. Others, however, can find no benefit from it. The active principle of it is horseradish. Aconite and chloroform have a similar effect. Has not found as good results from carvacrol as some have, especially in treating abscess. If we make a pet of a thing we can make it perform tricks for us that it will not for others; and much depends on efforts which we make which are other than physical.

Dr. W. H. Allen has used the "dental pain obtunder" without any results. It makes him positively sick. He wants none of it.

Dr. Stockton said that his patients complained of the smell of it. He has found no benefit from it whatever. He had used salicylic acid successfully. He thought a great deal of the trouble with dead teeth depends upon cleansing them too thoroughly at once, and he does not try to do it.

Dr. Osmond spoke of a preparation called "king of pain," which he was informed was oil of mustard. He has found oil of peppermint very useful for sensitive dentine. In fact, he had never found anything equal to it.

Prof. Flagg has tried all the various oils, and oil of peppermint was the worst thing to make patients howl he knew of; his experience with the "dental pain obtunder" was half-way between that of Drs. Taft and Allen. It does not seem to do much good and occasionally is exceeedingly painful. One remedy will do great things for one person and will do nothing for another. Pond's Extract is a very valuable medicine. In a case of shooting of the hand so badly that it was supposed that amputation would be necessary, the extract had been dropped upon the hand for some hours, and the result was the hand was saved. He is obliged to use allopathic and homœpathic remedies, and this is a quack remedy.

Dr. Shepard said that temperament made a great difference with the use of pain obtunders. The nervous effect is often a great deal. If you tell a patient that you are going to use some wonderful thing, and use only water, it would have a great effect. They don't have such sensitive dentine in Boston; there are only a few patients, mostly young, with whom we care for any obtunder. The miasmatic influences of certain localities render dental troubles more obstinate; they also aggravate inflammation. Has found benefit from the "dental pain obtunder." It often produced intense pain. In one mouth it seemed to do some good, and the same day in the same mouth it did no good; besides, it smelt badly. He is conducting some experiments, and thinks he is on the right track.

Dr. Morrison thought the knowledge of how to excavate, not taking too large chips, and with keen instruments, would succeed best. Had used thymol, etc., and thinks nothing superior to good old-fashioned creasote.

Dr. Knapp said that some agent would be a boon to those operating in miasmatic regions. The "dental pain obtunder" causes no more pain with him than any other cold substance. The teeth that are not sensitive in his region are the exception. Has found lime-water used for three or four weeks very beneficial. Is sometimes compelled to resort to blue pill, quinine, and iron before he could operate without danger of devitalizing the pulp, and he could not treat exposed pulps without so doing. We must eradicate specific poison before we can operate upon the teeth successfully.

Dr. Butler thought the electrical condition might have something to do with it, and perhaps the condition of the operator. The pain obtunder has a sickening odor, but in many cases he has found great benefit from it. Thought the coolness was the cause of the pain.

Dr. C. S. Smith had observed something like constitutional effects from the pain obtunder; it seemed to stupefy the whole system, and he thought it was a powerful drug of some kind and should be used with caution. He had heard it suggested that it was hasheesh.

Dr. Harlan said that it was oil of mustard, camphor, aconite, and ether.

The subject was then passed.

A discussion ensued upon the subject of clinics in connection with the future meetings of the society.

Dr. Abbott moved that we have no clinics.

Dr. Atkinson moved to amend by substituting a resolution of thanks to Drs. Bonwill, Webb, Gaylord, and Klump, who had contributed so much to the interest of the meeting by their operations at the clinic. The substitute was put to vote, and carried.

The newly-elected officers were then installed; and Dr. Northrop, the incoming president, made a few appropriate remarks.

A vote of thanks to the Executive Committee and Dr. Southworth, for their efforts to make the meeting a success, was adopted, and also to the hotels for reduced rates.

Dr. Keely, chairman of the committee on the Barnum testimonial fund, made a verbal report. He said the committee had hoped to be able to close up the matter and make a final report at this session, but they had not succeeded in getting returns from all the subscriptions that had been made. They held Dr. Barnum's receipt for ten hundred and seventy dollars, and had about one hundred dollars on hand; besides which there were unpaid subscriptions to the amount of about one hundred and fifty dollars, which he believed would all be paid up. He asked for another year to finish up the matter; which, on motion, was granted.

The chair appointed as the special committee of arrangements for the next year, Drs, McQuillen, Buckinham, and Pierce.

The association then adjourned to meet in Philadelphia on the first Tuesday of August, 1876.

---

